# Tyrosinase Inhibitors: A Perspective

**DOI:** 10.3390/molecules28155762

**Published:** 2023-07-30

**Authors:** Mason A. Baber, Cole M. Crist, Noah L. Devolve, James D. Patrone

**Affiliations:** 1 Department of Medicinal Chemistry, University of Michigan, Ann Arbor, MI 48209, USA; mabab@umich.edu; 2 Program in Biochemistry & Molecular Biology, Rollins College, Winter Park, FL 32789, USA; ccrist@rollins.edu; 3 Department of Chemistry, Rollins College, Winter Park, FL 32789, USA; ndevolve@rollins.edu

**Keywords:** tyrosinase, tyrosinase inhibitor, melanogenesis, natural products

## Abstract

Due to its integral role in the biosynthesis of melanin in all kingdoms of life, tyrosinase has become an extremely important target for inhibition in several sectors of research including agricultural and cosmetic research. Inhibitors of tyrosinase have made it to the market in the cosmetics industry, but their use has been limited due to conflicting efficacy and potential toxicity, which has led to several small molecules being removed from the market. Undaunted, researchers have continued to pursue tyrosinase inhibitors with varying degrees of success. These pursuits have built an impressive and rich library of research. This review is intended to provide a perspective of the past twenty years (2003–2023) of research on tyrosinase inhibitors by highlighting exemplar molecules and developments.

## 1. Introduction

**Figure 1:**
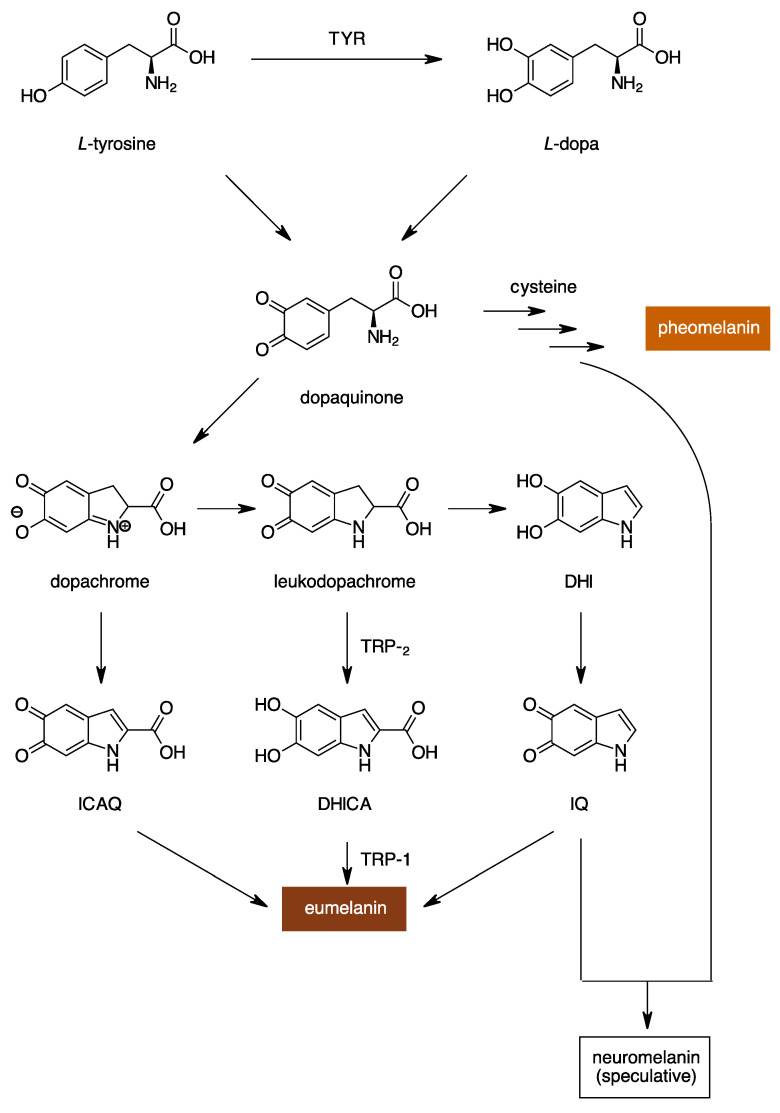
Melanogenesis pathway resulting in the formation of either eumelanin, pheomelanin, or neuromelanin (an understudied phenomenon). (TYR: tyrosinase; TRP: tyrosinase-related protein; L-dopa: L-3,4-dihydroxyphenylalanine; DHICA: 5,6-dihydroxyindole-2-carboxylic acid; DHI: 5,6-dihydroxyindole; ICAQ: indole-2-carboxylic acid-5,6-quinone; IQ: indole-5,6-quinone) [8,9,10].

**Figure 2:**
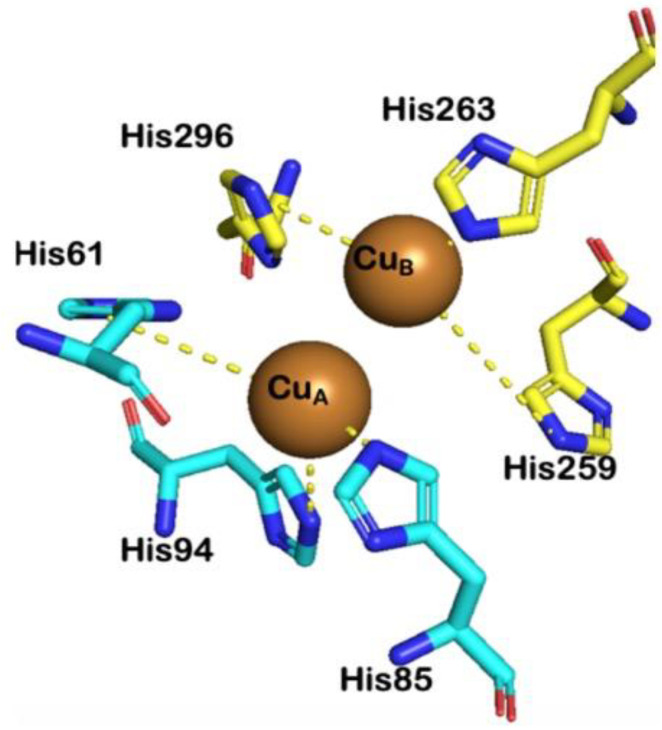
Unoccupied active site of TYR (PDB ID: 1BUG). Histidine residues coordinating to Cu_A_ and Cu_B_ are shown in blue and yellow, respectively [22].

Melanin is a biopolymer produced by melanogenesis, a nearly universal biosynthetic pathway that spans species and kingdoms. Melanogenesis begins with the oxidation of a monophenol (e.g., L-tyrosine) or a diphenol (e.g., L-dopa) to dopaquinone (Figure 1) [1,2,3]. Subsequent steps use dopaquinone to either make pheomelanin or eumelanin (red–yellow or black–brown in color, respectively). Recently, neuromelanin has been described as another form of melanin found in the brain and has properties of both pheomelanin and eumelanin, but its significance is not fully understood and is currently underexplored [4,5,6]. The initial oxidations of the pathway are both mediated by a type-III copper-containing oxidoreductase, tyrosinase (TYR, EC 1.14.18.1) [7,8,9,10,11,12]. After these oxidations, much of the remaining biosynthesis proceeds non-enzymatically. Thus, TYR is the key enzyme of melanogenesis as it catalyzes the rate-limiting steps of the entire pathway. Although TYR exhibits dual catalytic activity, the transformations occur in the same highly conserved active site, which is composed of six histidine residues coordinating two copper ions (Figure 2) [7,8,9,10,11,12]. The distinction in monophenolase or diphenolase activity arises from the presence or absence of a bridging water or hydroxyl molecule between the copper centers [8,9,10]. 

Due to its crucial role in melanogenesis, TYR has attracted attention in agronomy, medicine, and the cosmetic industry. In humans, melanin is responsible for pigmentation in our eyes, hair, and skin, which provides protection against UV radiation [13]. The overactivity or overexpression of TYR can result in hyperpigmentation disorders and has been associated with melanomas. In fruits and vegetables, TYR activity results in enzymatic browning or ripening. Therefore, TYR inhibitors could be useful for food preservation or as medicinal agents to treat disorders and diseases associated with the accumulation of melanin [8,14]. Numerous studies have identified both synthetic and natural inhibitors of TYR. However, many TYR inhibitors have not seen widespread use due to a lack of efficacy and/or toxicities such as carcinogenicity or cytotoxicity.

Interest in TYR and efforts to overcome the common challenges associated with TYR inhibitors have exponentially increased since the early 2000s, and recent advances in biochemical and biophysical methods have allowed us to better interrogate TYR biology [2,8,15,16,17,18,19,20]. Improved expression and purification protocols have made both the mushroom (mTYR) and human (hTYR) versions of the enzyme more readily available, which has enabled high-resolution crystal structures, rational structure-based design, and the optimization of inhibitors [21,22,23,24,25,26]. As the body of literature surrounding TYR is rapidly growing, we have curated and highlighted important discoveries and developments regarding TYR inhibitors from 2003–2023 to provide a digestible overview of the recent progress. Further, as this manuscript was intended to be a perspective, our goal was to highlight a breadth of chemotypes, discovery and optimization techniques, and molecules that have progressed the furthest toward the clinical setting. 

Based on the intense interest in TYR as an anti-melanogenesis target, there is substantive literature on the discovery and optimization of TYR inhibitors. A keyword search of “tyrosinase inhibitor” in Sci-Finder resulted in 1842 journal articles and PubMed resulted in 3699 journal articles for our targeted time frame. Within the Sci-Finder results, 1627 articles were written in English. The results were broken down by year, and the articles were accessed based on the availability of the journal article and evaluated. We focused our attention on three key areas: (1) molecules that have progressed furthest towards the clinic, (2) the potency of the in vitro IC_50_ value, and (3) the novelty of the technique. As such, we realize there are many deserving molecules, accomplishments, and scientists that are not covered here.

## 2. Highlighted Tyrosinase Inhibitors

**Figure 3:**
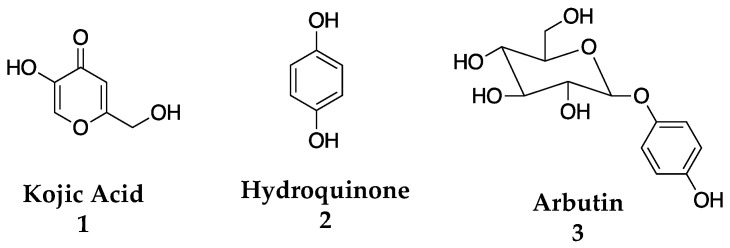
Previously identified commercial TYR inhibitors [9,30,31,32].

While the focus of this perspective is on the years 2003–2023, it is imperative to give context, and as such it must be noted that vast research on TYR inhibitors has occurred throughout the 20th century. Before 2003, there were many inhibitors discovered from both natural and synthetic sources from various research programs. Please see Seo et al. for a more thorough review of these molecules [9]. Around the turn of the century, research in the field was keenly interested in finding both more potent TYR inhibitors and novel chemical matter that possessed a better cytotoxicity profile [9,27,28,29]. Both synthetic molecules and natural products were mined for potential inhibitors. At the time of 2003, three molecules—kojic acid (**1**), hydroquinone (**2**), and arbutin (**3**)—were considered the gold standard of TYR inhibition and were used in cosmetic applications despite efficacy and toxicity issues (Figure 3) [9,30,31,32]. 

### 2.1. 2003

**Figure 4:**
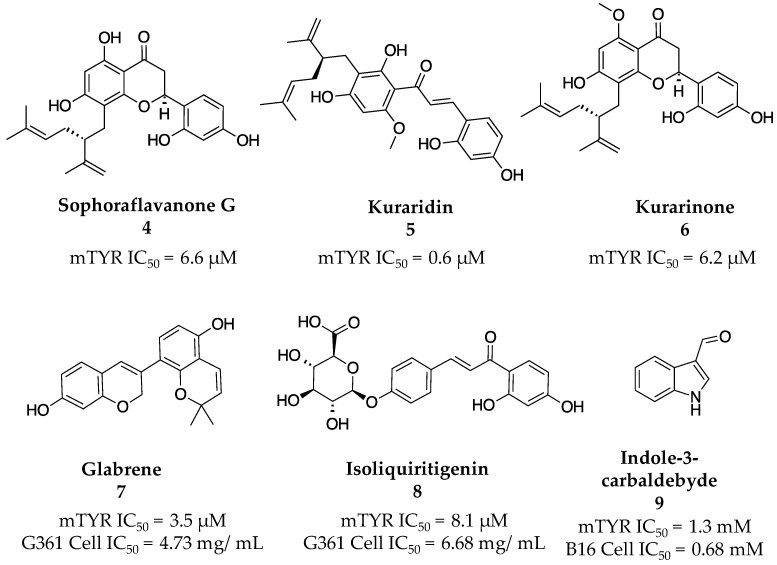
Exemplar inhibitors from 2003 [33,34,35].

Material from the shrub *S. flavescens* had previously displayed TYR inhibitory activity and thus had the potential for further inhibitor discovery [33]. The Kim group was able to isolate three known prenylated flavonoids—sophoraflavanone G (**4**), kuraridin (**5**), and kurarinone (**6**)—from successive ethanol and dichloromethane column chromatography purifications (Figure 4). Though these molecules had been previously isolated and described in the literature, there were no reports of their TYR inhibition. In this case, the researchers tested the isolated compounds’ inhibitory capacity against mTYR and found IC_50_ values of 6.6, 0.6, and 6.2 µM for sophoraflavanone G (**4**), kuraridin (**5**), and kurarinone (**6**), respectively.

Nerya et al. then investigated the inhibitory effects of licorice root extracts on TYR activity. They found that the extract inhibited activity more substantially than galibridin, an already established and potent TYR inhibitor. [34]. Further investigation led to the discovery of glabrene (**7**) and isoliquiritigenin (**8**), which inhibited the monophenolase activity of TYR with IC_50_ values of 3.5 and 8.1 µM, respectively (Figure 4). The authors also characterized the inhibitors in terms of their kinetic mechanisms and elucidated that both molecules were mixed inhibitors and possessed K_i_ values of 180 and 990 µM in regard to their inhibition of monophenolase activity. Additional work showed that glabrene (**7**) and isoliquiritigenin (**8**) possessed IC_50_ values of 4.73 and 6.68 mg/mL against melanin production in G361 human melanocytes with minimal cytotoxicity.

The Shimizu research group investigated the inhibitory effects of extracts from the novel fungus YL185 [35]. Following treatment of the extracellular liquid with ethyl acetate, indole-3-carbaldehyde (**9**, Figure 4) was found to be the active constituent within the soluble fraction. Aldehyde **9** had a modest IC_50_ value of 1.3 mM against mTYR but inhibited melanin synthesis at 0.68 mM without significant cytotoxicity in B16 melanoma cells.

### 2.2. 2004

**Figure 5:**
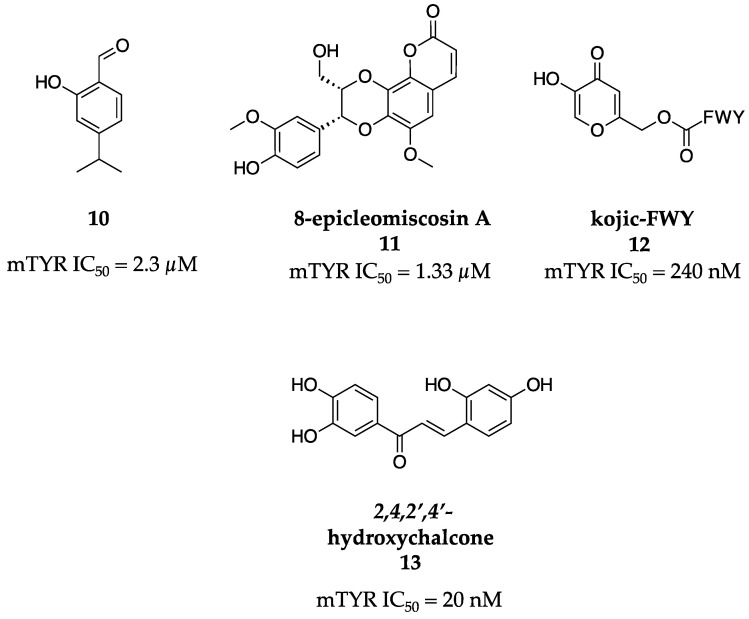
Exemplar inhibitors from 2004 [36,37,38,39].

To expand upon the success of plant-based TYR inhibitors, the Kubo research group synthesized 2-hydroxy-4-isopropylbenzaldehyde (**10**, Figure 5) [36]. A kinetic analysis of 10 proved that it was a partial inhibitor of the diphenolase activity of mTYR with an IC_50_ of 2.3 µM. Further, a Dixon plot revealed that it was mixed inhibitor. The authors speculated that this may be due to the aldehyde group forming a Schiff base with a primary amine group on the enzyme near the active site, where it does not totally exclude substrate binding, making the molecule only a partial inhibitor.

Ahmad et al. isolated five compounds from *Rhododendron collettianum*, a flowering plant abundant in the region between Pakistan and Afghanistan [37]. The most promising compound was a novel coumarinolignoid, 8-epicleomiscosin A (**11**, Figure 5). 8-Epicleomiscosin A (**11**) was isolated through chloroform extraction of the aerial parts of the plant following column chromatography. Impressively, 8-epicleomiscosin A (**11**) showed an IC_50_ value of 1.33 µM against mTYR diphenolase activity.

In an effort to investigate if kojic acid (**1**) could be derived to maximize its potency and lower its cytotoxicity, Kim and colleagues investigated the effects of adding a tripeptide moiety to kojic acid (**1**) [38]. Solid-phase synthesis using a trityl-based bead and standard F-moc peptide synthesis was employed to synthesize five novel analogs. The best of these molecules was kojic-FWY (**12**, Figure 5), which possessed an IC_50_ value of 240 nM for monophenolase activity against mTYR and showed no cytotoxicity at 100 ppm in cultured murine melanocytes (melan-a cells). Both the gain in potency and lack of cytotoxicity represented significant progress when compared to kojic acid (**1**) alone.

The Vaya research group was interested in leveraging the significant amount of literature on the 4-substituted resorcinol moiety by synthesizing five molecules including 2,4,2′,4′-hydroxychalcone (**13**, Figure 5) [39]. This molecule was asymmetrical, yet it possessed two different 2,4-substituted phenols, which were postulated, and proven, to be extremely important for binding, with monophenolase and diphenolase IC_50_ values of 20 nM and 90 µM against mTYR, respectively. This was the most potent inhibitor to date of mTYR.

### 2.3. 2005

**Figure 6:**
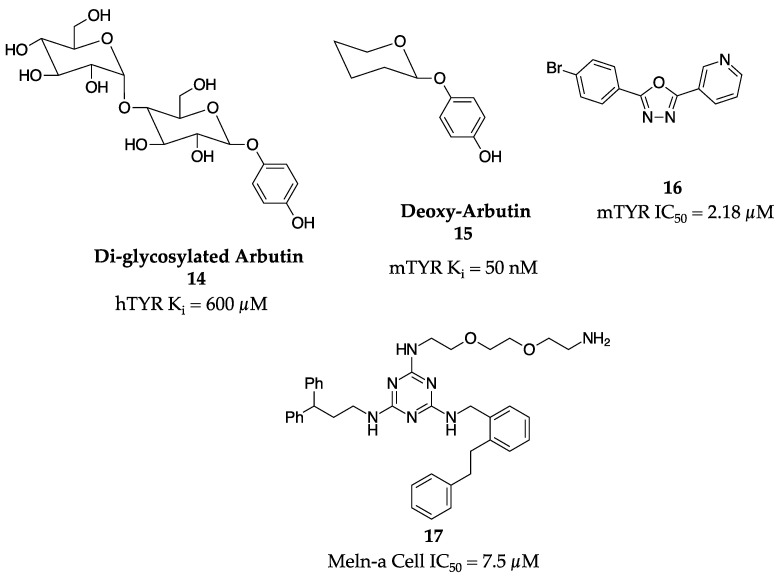
Exemplar inhibitors from 2005 [40,41,42,43].

Two research groups used similar but novel strategies in trying to optimize the known inhibitor arbutin (**3**). In Japan, the Sugimoto group used a chemoenzymatic approach to transglycosylate -arbutin with CGTase from *B. macerans* to form both a mono- and di-glycosylated analog of arbutin (**14**, Figure 6). [40] The mono-glycosylated analog showed a K_i_ value of 600 µM against the diphenolase activity of hTYR. Concurrently, Boissy and colleagues took the opposite approach of tailoring arbutin (**3**) down in size with great success [41]. The researchers identified deoxyarbutin (**15**, Figure 6) as a powerful inhibitor with potential for use in humans. The K_i_ value for monophenolase activity against mTYR was 50 nM, far more potent than hydroquinone (**2**) or arbutin (**3**), which prompted further analysis. Deoxyarbutin (**15**) was taken forward to both a guinea pig model and a 12-week human clinical trial. In both these studies, deoxyarbutin (**15**) performed admirably with sustained and reversible skin lightening in the animal model and a slight to significant reduction in overall skin lightness in humans.

The Khan group, which previously had identified natural product-based TYR inhibitors, took a one-step microwave-assisted synthetic approach to build a library of twenty-six oxadiazoles [42]. Of these molecules, eighteen had single-digit micromolar IC_50_ values against the diphenolase activity of mTYR. The best analog (**16**, Figure 6) had an IC_50_ value of 2.18 µM and contained a 3-pyridyl substitution, which was important for potency most likely by helping to chelate the copper atom in the active site, and a 4-position bromine on the other phenyl ring. 

A novel cell-based screening approach to identify TYR inhibitors was used by Ni-Komatsu et al [43]. A 1535-membered tagged triazine library was screened in melan-a cells for the inhibition of melanin. From this screening, four molecules were identified, with triazine (**17**) being the most potent with an IC_50_ in melan-a cells of 7.5 µM (Figure 6). Triazine (**17**) and other molecules were found to be competitive inhibitors that increased the lag phase of TYR in in vitro studies. Further, these molecules were proven to be safe as there were no morphologic changes or obvious toxicity at a concentration of 20 µM in the melan-a cells.

### 2.4. 2006

**Figure 7:**
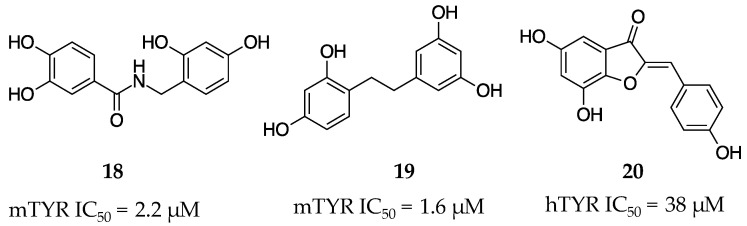
Exemplar inhibitors from 2006 [44,45,46].

The Park Lab designed a novel series of TYR inhibitors, N-benzylbenzamides, by grafting a polyphenolic catechol or a 2,4-dihyroxyphenyl group onto the basic structure of the molecule chalcone with an amide linker [44]. The authors synthesized twenty phenol derivatives and tested them against the diphenolase activity of mTYR. The best molecule (**18**, Figure 7) was substituted with 3,5-OH groups on benzene ring A and 2′,4′-OH on benzene ring B. The molecule was later kinetically characterized as a mixed inhibitor that possessed an IC_50_ value of 2.2 µM.

Using the known TYR inhibitor oxyresveratrol as a template, Likhitwitayawuid et al. created a series of analogs to fine-tune the molecule’s inhibitory activity and cytotoxicity [45]. The authors were able to both improve TYR inhibitory potency and abrogate toxicity via hydrogenation of the central double bond to fully saturate analog **19** (Figure 7). Molecule **19** was a non-competitive inhibitor with an IC_50_ of 1.6 µM and K_i_ of 4.8 µM. Beyond its potency, **19** was also shown to have no cytotoxicity in KB, BC, and NCI-H187 cell lines.

Boumendjel’s group assessed both naturally occurring and synthetic aurones (*Z*-benzylidenebenzofuran-3(2H)-one) as TYR inhibitors [46]. Of the molecules tested, the best compound was the natural analog **20** (Figure 7). Aurone (**20**) possessed an IC_50_ of 38 µM and 75% inhibition of melanin production in melanocytes at 0.1 mM and was chosen as the lead for further study and progression. With the goal of using **20** in humans, experiments performed on rats revealed no toxicity following oral administration at a dose of 5 g/kg with no significant irritation following topical or eye dosage in rabbits. Even more promising, **20** showed no difference in melanin inhibition in melanocytes from people of different color (white, dark, and black skin) at either concentration tested. 

### 2.5. 2007

**Figure 8:**
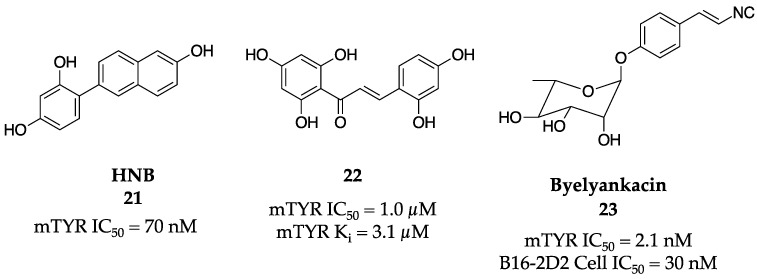
Exemplar inhibitors from 2007 [47,48,49].

Based on the previously highlighted effectiveness of oxyresveratrol, it was mined further as a template for novel analogs that could inhibit TYR. The Chung group designed a group of new hydroxyl-substituted phenyl naphthalenes to mimic the hydroxy and phenyl groups of oxyresveratrol [47]. Their exemplar molecule was 4-(6-hydroxy-2-naphthyl)-1,3-bezendiol (HNB, **21**, Figure 8), which was found to be a competitive inhibitor with an IC_50_ value of 70 nM against mTYR. It also inhibited melanin production in B16F10 melanoma cells with no cytotoxicity found. Additionally, the potential of HNB (**17**) as an efficacious TYR inhibitor warranted the authors to file a patent for the molecule (application number 10-2006-0071433).

Jun et al. later gained interest and were successful in developing a new class of TYR inhibitors using chalcone as their inspiration [48]. Based on the privileged chalcone pharmacophore, the researchers synthesized a library of various, novel hydroxychalcones and found that the addition of a trihydroxy moiety was the best (**22**, Figure 8). Molecule **22** was shown to be a competitive inhibitor with an IC_50_ value of 1 µM and a K_i_ value of 3.1 µM against the diphenolase activity of mTYR.

The Nakagawa research group identified byelyankacin (**23**, Figure 8) as a novel melanogenesis inhibitor after it was isolated from *Enterobacter* sp. B20 [49]. Byelyankacin (**23**) possesses an α-L-rhamnopyranoside, phenyl ring, and an isocyanovinyl group. After characterization, byelyankacin (**23**) was shown to inhibit mTYR and the melanogenesis of B16-2D2 melanoma cells with IC_50_ values of 2.1 nM and 30 nM.

### 2.6. 2008

**Figure 9:**
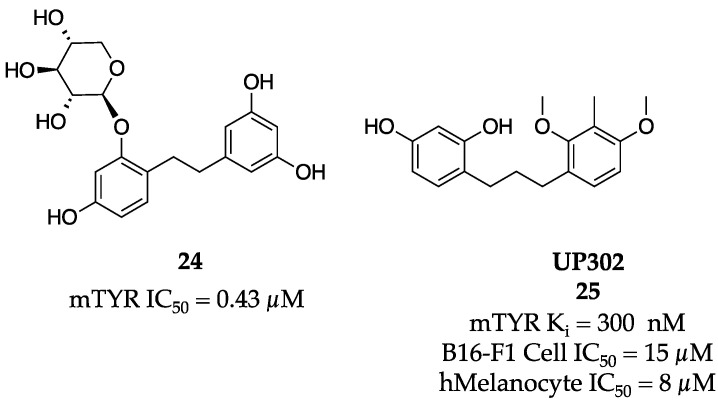
Exemplar inhibitors from 2008 [50,51].

The bibenzyl pharmacophore was further explored via a four-membered synthetic library by Oozeki et al. [50]. Looking to improve on the naturally occurring pharmacophore, a short synthesis utilizing a Wittig reaction and trichloroimidate glycosylation was implemented. Bibenzyl (**24**) was found to be the most potent inhibitory molecule with an IC_50_ value of 0.43 µM (Figure 9).

Nesterov et al. screened 1144 plant extracts and were able to identify twenty which potently inhibited mTYR [51]. Three of the identified compounds contained a diarylpropane structure including UP302 (**25**, Figure 9). As UP302 (**25**) was not appreciably abundant in its natural source, a synthesis was designed, and the molecule was evaluated thoroughly. UP302 (**25**) was found to competitively inhibit the diphenolase activity of mTYR with a K_i_ value of 300 nM. Using melanoma cells B16-F1 and human primary melanocytes, UP302 (**25**) inhibited melanin formation with IC_50_ values of 15 and 8 µM, respectively. Of significant importance, UP302 (**25**) applied at 0.1% in a reconstructed skin model displayed significant skin lightening with no effect on cell viability or morphology.

### 2.7. 2009

**Figure 10:**
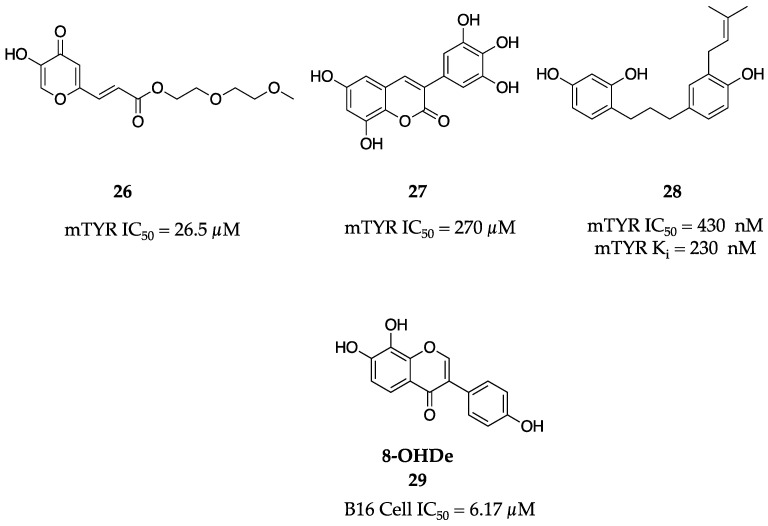
Exemplar inhibitors from 2009 [52,53,54,55].

Hoping for a synergistic effect, Kang et al. merged pieces of the known TYR inhibitors kojic acid (**1**) and caffeic acid into a hybrid series [52]. This series of molecules was synthesized via a Horner–Wadsworth–Emmons reaction followed by derivatization. Molecule **26** proved to be the most efficient inhibitor of mTYR monophenolase activity possessing an IC_50_ of 26.5 µM (Figure 10). Further experiments showed that **26** lowered melanin production in B16F10 mouse melanoma cells at 20 µg/mL and had a cell viability of 84.6% at this concentration.

In a similar hybrid strategy, the Delogu research group synthesized a library of coumarin–resveratrol hybrids to investigate the SAR governing their anti-tyrosinase activity [53]. The synthesized derivatives varied in the amount and position of hydroxyl substituents in the three-ring system, with the lead compound **27** showing the greatest potency, inhibiting mTYR in a non-competitive fashion with an IC_50_ value of 270 µM (Figure 10).

Six 1,3-diphenylpropane molecules were isolated and characterized from the methanol extract of the deciduous shrub *Broussonetia kazinoki* [54]. These molecules were tested for TYR inhibition and molecule **28** was identified as the most potent (Figure 10). A rigorous kinetic profile showed that **28** was a simple, reversible, slow-binding competitive inhibitor with an IC_50_ of 430 nM and K_i_ of 230 nM for monophenolase activity as well as an IC_50_ of 570 nM and K_i_ of 290 nM for diphenolase activity of mTYR.

Shou-Ku Tai at el. investigated the in vivo anti-tyrosinase activity of 8-hydroxydaidzein (8-OHDe, **29**, Figure 10), a molecule known to inhibit mTYR in an irreversible manner in vitro [55]. Irreversible or suicide inhibitors of TYR are scarcely reported in the literature compared to reversible inhibitors and as such, exploring the potential of **29** in vivo was a critical step forward. 8-OHDe (**29**) was shown to inhibit TYR activity in B16 melanoma cells with an IC_50_ of 6.17 µM and was not cytotoxic. This result prompted the researchers to test 8-OHDe (**29**) in a 2% and 4% treatment on human volunteers and found a significant increase in δL*-values after three weeks. Thus, 8-OHDe (**29**) was labeled an excellent candidate to move forward as a skin-whitening agent.

### 2.8. 2010

**Figure 11:**
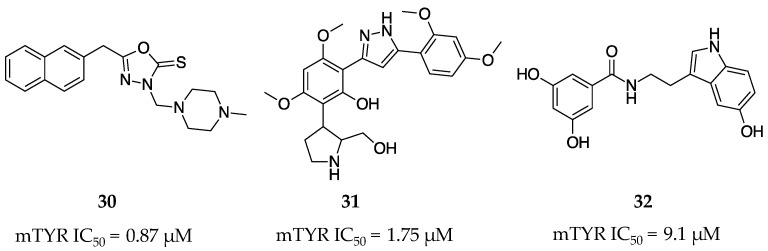
Exemplar inhibitors from 2010 [56,57,58].

Lam et al. synthesized a library of sixteen oxadiazole and triazolothiadiazole derivatives that were observed to display potent inhibition of mTYR [56]. The lead compound **30** was a triazolothiadiazole containing a piperazine ring that was found to inhibit mTYR with an IC_50_ of 0.87 µM (Figure 11). Crystal structures later showed that the inhibition of TYR by **30** was dependent on the chelation of a thiocarbamate sulfur to the dicopper active site. 

Bandgar et al. devised a synthetic route to produce a combinatorial library of 3,5-diaryl pyrazole derivatives to investigate their anti-cancer and anti-inflammatory properties [57]. In their studies, it was found that the lead compound **31** was able to competitively and potently inhibit the mono- and diphenolase activity of mTYR with IC_50_ values of 1.75 and 2.84 µM, respectively (Figure 11). Compound **31** was also found to be non-toxic in CCK-8 cell lines. 

*N*-(3,5-Dihydroxybenzoyl)-6-hydroxytryptamine (**32**, Figure 11) was synthesized by Yamazaki and Kawano as a novel inhibitor of TYR [58]. Compound **32** was reported to irreversibly inhibit TYR diphenolase activity against HMV-II hTYR melanoma cells with an IC_50_ of 9.1 µM, which was significantly more potent than kojic acid (**1**, IC_50_ = 310 µM). The potent and irreversible behavior of **32** was observed at low concentrations of L-dopa, but this effect was attenuated at increasing substrate concentrations. Unfortunately, **32** did not show a reduction in melanin production in HMV-II hTYR melanoma cells and caused cell viability issues at the concentrations tested.

### 2.9. 2011

**Figure 12:**
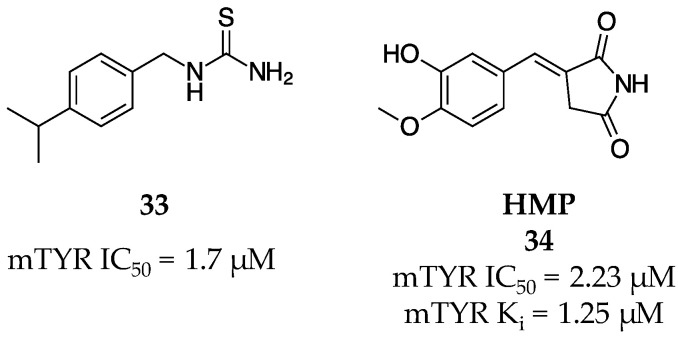
Exemplar inhibitors from 2011 [59,60].

Thanigaimalai et al. synthesized and evaluated a library of 1-phenylthioureas and 1,3-disubstituted thioureas in an attempt to examine the inhibitory effects of additional phenyl groups and various phenylic substituents on the known potent inhibitor of TYR, phenylthiourea (PTU, IC_50_ = 1.8 µM) [59]. This library showed that various alkyl substituents at the 2- through 4-position, as well as a singular methylene connecting group between the phenyl and ureal functional groups resulted in the highest potency with respect to TYR inhibition. In a biochemical assay, the 1-substituted thioureas were shown to be TYR inhibitors, whereas the 1,3-disubstituted thioureas did not display anti-tyrosinase activity despite inhibiting melanogenesis in cells. The best TYR inhibitor (**33**, Figure 12) had a 4-position isopropyl substitution, an IC_50_ of 1.7 µM against mTYR and 95% inhibition of melanin at 10 µM in B16 cells.

A novel chemical family of hydroxybenzylidenyl pyrrolidine-2,5-diones was identified as potent TYR inhibitors by the Chung research group [60]. The best molecule (HMP, **34**, Figure 12) had an IC_50_ of 2.23 µM for mTYR monophenolase activity. A further kinetic analysis revealed HMP (**34**) to be a competitive inhibitor with a K_i_ of 4.24 nM at 1.25 µM and a K_i_ of 1.82 µM at 20 µM. In silico docking using DOCK6 showed that **34** directed the phenol group into the active site to interact with the copper centers, interacted with a suite of amino acids including Val93, Pro96, Thr97, Trp239, Leu297, Pro323, Asp324, Gly328, and Lys329 with Thr97 being critical to binding, and had a predicted binding energy of −31.67 kcal mol^−1^. Molecule **34** showed promising cellular melanogenesis inhibition and no cytotoxicity up to 10 µM. 

### 2.10. 2012

**Figure 13:**
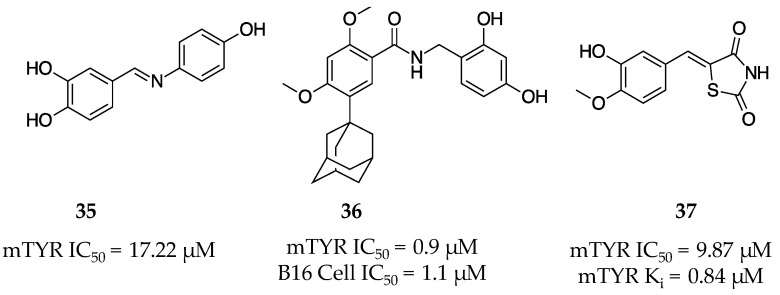
Exemplar inhibitors from 2012 [61,62,63].

Bae et al. designed and synthesized multiple (*E*)-*N*-substituted benzylidene-hydroxy and methoxy-aniline analogs [61]. These compounds were examined in vitro for their TYR inhibition against the monophenolase activity of mTYR as well as anti-melanogenesis activities in B16F10 melanoma cells. It was reported that (*E*)-4-((4-hydroxyphenylimino)methyl)benzene-1,2-diol (**35**, Figure 13) was the most potent inhibitor synthesized with an IC_50_ = 17.22 µM against mTYR, and it operated via a non-competitive mechanism. The cellular activity of **35** was quite promising as it significantly inhibited melanogenesis at all concentrations without any cytotoxicity issues. The authors proposed that due to the TYR inhibitory activity of **35**, as well as its ability to decrease melanin production in melanoma cells in a dose-dependent manner, this small molecule possesses the potential to be explored as a therapeutic agent. 

Baek and colleagues synthesized a library of eleven polyphenolic *N*-benzyl benzamides and examined their potential as inhibitors against mTYR and melanogenesis inhibitors in the B16 melanoma cell line [62]. It was found that specific increases in hydrophobic R-groups originating from the various phenyl rings increased potency and decreased cytotoxicity by a noticeable margin, and one of the small molecules synthesized (**36**, Figure 13) exhibited a high potency for TYR inhibition (IC_50_ = 0.9 µM) and melanogenesis inhibition (IC_50_ = 1.1 µM) and a relatively low cytotoxicity (95% viability at 10 µM after 75 h). Compound **36** was a good candidate for a skin-whitening agent as it proved to maintain a low cytotoxicity comparable to that of the kojic acid (**1**) control, while maintaining a nearly 500-fold more efficient inhibition of melanogenesis. 

A library of twelve 5-(substituted benzylidene)thiazolidine-2,4-diones was rationally designed and synthesized based upon the known structural characteristics of tyrosinase’s natural substrate L-dopa and known inhibitor PTU. Upon examining the compounds for their potential as TYR inhibitors, (*Z*)-5-(3-hydroxy-4-methoxybenzylidene)thiazolidine-2,4-dione (**37**, Figure 13) was reported to have a much higher potency (IC_50_ = 9.87 µM) than the positive control, kojic acid (**1**), against the monophenolase activity of mTYR. Molecule **37** was found to be the most efficacious inhibitor through kinetic analysis with K_i_ value 0.84 µM at 20 µM and operated in a competitive manner. The binding mode of **37** was interrogated through docking simulations with potato catechol oxidase using DOCK6 to identify potential amino acid contacts and a predicted binding energy of 27.46 kcal mol^−1^. Further, **37** inhibited the production of melanin in B16 melanoma cell lines [63].

### 2.11. 2013

**Figure 14:**
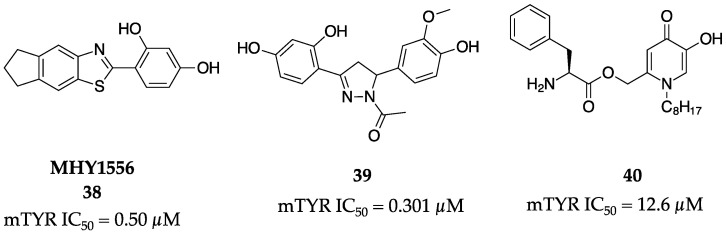
Exemplar inhibitors from 2013 [64,65,66].

Park et al. synthesized a de novo TYR inhibitor, MHY1556 (**38**, Figure 14) [64]. Compound **38** exhibited a superior inhibitory effect (IC_50_ = 0.50 µM) compared to the positive control kojic acid (**1**, IC_50_ = 53.95 µM) in vitro against mTYR. Furthermore, the authors used TYR tertiary structural predictions with the QMEAN server along with in silico docking studies to assess the binding mode and found a predicted binding energy of 7.03 kcal/mol via AutoDock4.2. These data suggested the importance of para- and ortho-hydroxyl substituents on the phenyl ring of the compound, granting insight into the structural significance of this molecule. MHY1556 (**38**) also proved to inhibit melanogenesis in B16F10 melanoma cells and lacked significant cytotoxicity in cell viability assays. 

A library of twenty 3,5-diaryl-4,5-dihydro-1H-pyrazole derivatives was synthesized and examined for inhibitory activity against mTYR by Zhou et al [65]. It was reported that among the compounds synthesized, the most potent inhibitor was **39** with an IC_50_ = 0.301 µM (Figure 14). A kinetic study of the compounds indicated that they all acted as competitive inhibitors. This study also granted insight into the structurally significant functional groups on these molecules that promote more potent inhibition, such as the 2,4-dihydroxy substituents on ring A and a 4-hydroxy substituent on ring B. 

Li and colleagues synthesized a library of kojic acid derivatives made of hydroxypyridinone-L-phenylalanine conjugates [66]. When evaluated for their inhibitory activity against mTYR, it was reported that compound **40** maintained the highest potency against both monophenolase and diphenolase activity with IC_50_ values of 12.6 and 4.0 μM, respectively (Figure 14). Through kinetic studies, it was found that this molecule acted as a mixed-type inhibitor, binding to both the enzyme–substrate complex as well as the free enzyme. Cell viability assays also confirmed that many of the compounds synthesized in this study were non-toxic to multiple cell lines. 

### 2.12. 2014

**Figure 15:**
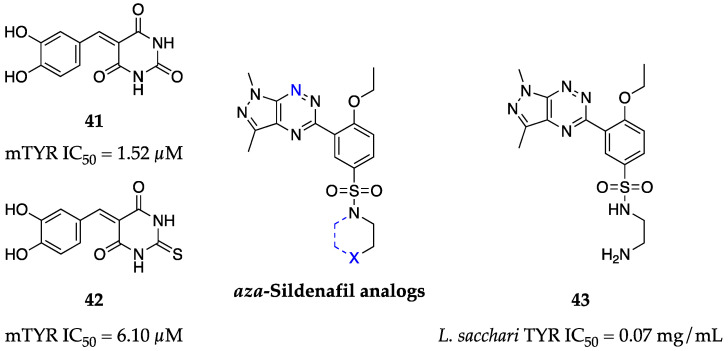
Exemplar inhibitors from 2014 [67,68].

Although barbituric acid and dihydroxybenzaldehyde do not themselves exhibit inhibitory activity against mTYR, Chen et al. observed that by coupling these scaffolds together, low micromolar inhibitors could be achieved [67]. Through systematic SAR work, Chen et al. also demonstrated the greater importance of the 4-hydroxy substituent than the 3-hydroxy substituent and the ablation of activity when these hydroxy groups were methylated. Their most potent compound (**41**, Figure 15) sported a 3,4-dihydroxy catechol pendant stemming from a barbituric acid core (IC_50_ = 1.52 µM) and was roughly 10-fold more potent than kojic acid (**1**). Similarly, the thiobarbituric acid analog **42** maintained a 3-fold improvement over kojic acid (**1**), but was slightly less potent than **41**.

Sildenafil, a PDE5 inhibitor, has a known interaction with TYR. To capitalize on this known off-target effect, Mojzych et al. proposed *aza*-analogs of sildenafil to retain the overall azoloazine core while reducing affinity for PDE5 through the removal of the carbonyl group [68]. In doing so, **43** was developed, exhibiting a 96% inhibitory rate (IC_50_ = 0.07 mg/mL) for bacterial TYR at 0.30 mg/mL with completely ablated inhibitory activity for PDE5 (0% inhibition at 1 µM) (Figure 15). Interestingly, this series departs from the common phenolic motifs of other TYR inhibitors and instead may rely on interactions between the active site coppers and the diamino side chain and π-electrons of the pyrazolotriazine core. 

### 2.13. 2015

**Figure 16:**
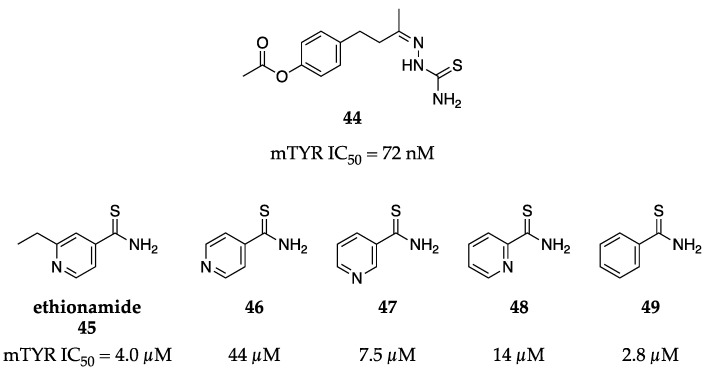
Exemplar inhibitors from 2015 [69,70].

Thiosemicarbazones are known metal chelators and, as a result, have become attractive motifs to include in the design of TYR inhibitors given their ability to coordinate to the copper ions of the active site. You et al. were some of the earliest adopters of thiosemicarbazone scaffolds and quickly achieved sub-µM inhibitory activity against mTYR [69]. Their SAR campaign revealed that addition to the terminal end of the thiosemicarbazone abolished activity, but addition to the phenol of the phenethyl pendant generally improved potency. Small, hydrophobic ethers were well-tolerated, but a simple acetoxy moiety proved to be the most optimal group, resulting in a nearly 400-fold more potent inhibitor than kojic acid (**1**) with an IC_50_ = 72 nM for diphenolase activity (**44**, Figure **16**). 

In a similar vein, the established TYR inhibitor PTU is known to chelate the active site copper ions [70]. To find structurally similar compounds, Choi et al. employed chemoinformatic methods (namely Tanimoto coefficients, T_c_) to screen a library of FDA-approved drugs [70]. Acetanilide was the most similar hit (T_c_ = 0.48) but was inactive against TYR. Interestingly, the replacement of the carbonyl in acetanilide with a thionyl resulted in the low µM inhibition of mTYR activity. This observation led Choi et al. to investigate ethionamide, an approved second-line antituberculosis drug, and its analogs (**45–49**, Figure 16). Although ethionamide has a low similarity score when compared to PTU (T_c_ = 0.21), it was equipotent (IC_50_ = 4.0 µM) to thioacetanilide (IC_50_ = 4.1 µM) and comparable in potency to PTU (IC_50_ = 1.3 µM). Preliminary SAR studies revealed even minor alterations to ethionamide could result in drastic potency changes. The removal of the ethyl group caused a 10-fold decrease in potency (**46**, IC_50_ = 44 µM), but pyridyl isomers **47** and **48** regained potency (IC_50_ = 7.5 µM and 14 µM, respectively). The most potent compound from their campaign was a simple thiobenzamide (**49**, IC_50_ = 2.8 µM), which was still 2-fold less potent than PTU in an enzymatic assay. In melanoma cells, thioacetanilide (**48**) and thiobenzamide (**49**) reduced melanin content with comparable potency to PTU at a 50 µM dose. Given these results, thiobenzamide (**49**) may serve as a simple starting point from which structure-based drug design could lead to a more potent series while addressing other limitations of ethionamide (**45**) or PTU.

### 2.14. 2016

**Figure 17:**
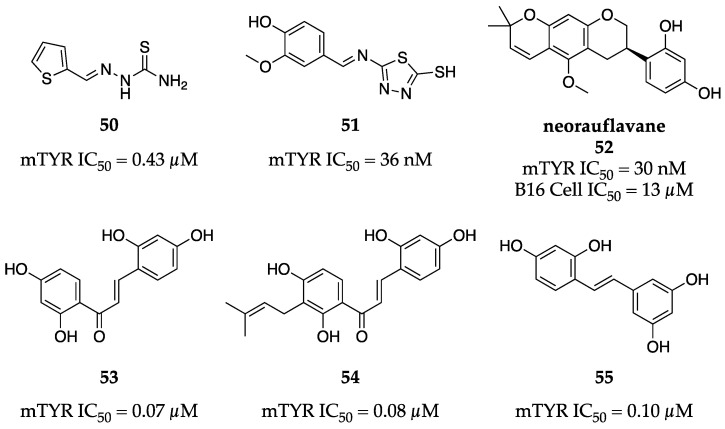
Exemplar inhibitors from 2016 [71,72,73,74].

Thiosemicarbazones and similar Schiff bases have continued to be popular among synthetic TYR inhibitors. Xie et al. further elaborated on the SAR campaign described by You et al. through the introduction of a heterocycle in place of the parent phenethyl pendant [71,72]. The heteroatom identity greatly influenced inhibitory activity. A basic pyrrole exhibited the weakest inhibition of the set (IC_50_ = 3.99 µM), whereas the electron-rich thiophene **50** demonstrated sub-µM inhibitory activity against mTYR (IC_50_ = 0.43 µM) (Figure 17). Similarly, Tang et al. found thiadiazoles bearing Schiff bases exhibited excellent inhibition of mTYR while exploring more decorated phenyl pendants compared to earlier work [69,72]. Exemplar **51** from their work impressively inhibited mTYR enzymatic activity with an IC_50_ of 36 nM.

Phenolic natural products isolated from various plant sources have continued to emerge as potent TYR inhibitors. For example, neorauflavane (**52**, Figure 17) was isolated from *Campylotropis hirtella* by Tan et al. and competitively inhibited monophenolase (IC_50_ = 30 nM) and diphenolase activity (IC_50_ = 500 nM) of mTYR [73]. Despite this potent inhibition in enzymatic assays, neorauflavane (**52**) reduced melanin content in B16 melanoma cells with a more modest IC_50_ of 13 µM. Zhang et al. isolated and identified several chalcones and a stilbene derivative from twigs of *Morus alba* L., which exhibited sub-µM inhibition of mTYR activity [74]. Chalcones **53** and **54** were equipotent inhibitors (IC_50_ = 0.07 µM and 0.08 µM, respectively), while stilbene **55** was slightly less potent (IC_50_ = 0.10 µM), but was reminiscent of resveratrol (Figure 17). 

### 2.15. 2017

**Figure 18:**
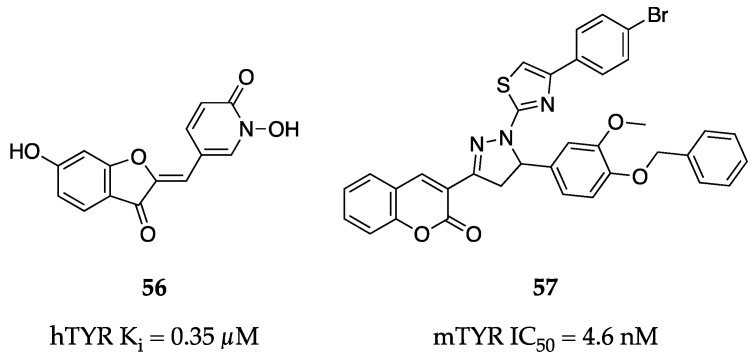
Exemplar inhibitors from 2017 [75,76].

As new protocols for expressing and purifying hTYR have emerged, researchers have been able to employ more physiologically relevant assay systems to assess novel inhibitors. For example, Haudecoeur et al. identified aurone derivatives sporting hydroxypyridine-*N*-oxide (HOPNO) pendants with low to sub-µM inhibition constants against purified hTYR [75]. The aurone core appeared to contribute significantly to binding and inhibition as HOPNO alone was a very weak inhibitor (K_i_ = 128 µM). The non-oxidizable HOPNO moiety is likely anionic at physiological pH, allowing a strong chelating interaction to occur with the copper centers of the active site while the dihydrobenzofuran core aids in binding through hydrophobic interactions and hydrogen bonding. The positioning of the phenol was significant as the 6′-OH derivative (**56**, Figure 18) demonstrated superior potency compared to other analogs (K_i_ = 0.35 µM for **56**, 3-fold improvement over analogs). Unfortunately, this HOPNO-aurone series experienced dramatic reductions in potency when administered to human melanoma whole cells and lysates. The potential zwitterionic nature of the HOPNO group likely causes these compounds to suffer from poor permeability, greatly limiting their use. However, it is worth noting this work by Haudecoeur et al. produced one of the most active hTYR inhibitors to date, which could still be used as a valuable chemical probe.

One of the most potent compounds against mTYR disclosed to date is **57** from the work of Saeed et al. (Figure 18). [76]. Their pyrazolinylthiazole compounds all demonstrate impressive sub-µM inhibition, but **57** reaches into the realm of single-digit nM (IC_50_ = 4.6 nM)—nearly 3700-fold more potent than kojic acid (**1**) [76]. Based on kinetic studies and a docking model, **57** appears to be a non-competitive inhibitor capable of blocking the binuclear copper active site. Importantly, the carbonyl of the chromanone pendant forms hydrogen bonds with two residues on the edge of the active site and positions the rest of the molecule to hinder access to the catalytic cavity. However, without a solved co-crystal structure to validate these models, the exact binding mode cannot be known.

### 2.16. 2018

**Figure 19:**
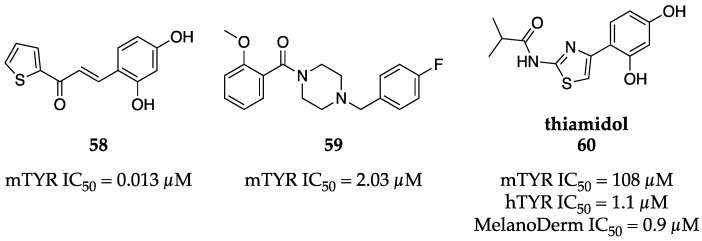
Exemplar inhibitors from 2018 [77,78,79].

Natural and synthetic chalcones appear frequently throughout the TYR literature. Their potencies vary, but many exhibit low to sub-µM inhibition of mTYR. Synthetic chalcone **58** prepared by Kim et al. is a potent inhibitor of both monophenolase and diphenolase activity (IC_50_ = 0.013 µM and 0.93 µM, respectively) (Figure 19) [77]. As one may expect, the location and number of substituents on the phenyl ring greatly influences the inhibitory activity. The 2,4-dihydroxy resorcinol motif is far superior to other analogs and molecular docking studies have attributed much of its activity to hydrogen bonding between the 2-hydroxy and an Asn residue of the active site. Kim et al. also investigated the effect of **58** in murine melanoma cells and observed a return to basal melanin levels when **58** was administered at 0.2 µM or 1 µM, even under melanin-stimulating conditions. 

Although there is a paucity of hTYR crystal structures, dozens of high-resolution structures are available for mushroom and bacterial TYR. These have allowed groups to employ structure-based drug design and rationalize the binding modes of novel inhibitors. For instance, Ferro et al. previously reported a new class of mTYR inhibitor bearing a 4′-fluorobenzyl moiety which, surprisingly, was the copper-binding domain of the inhibitor as indicated by co-crystallization [78]. More specifically, the aromatic ring establishes π–π interactions with an active site His residue. Using this observation, Ferro et al. designed arylpiperidine and arylpiperazine series bearing 4′-fluorobenzyl moieties as the key recognition component. Further elaboration on this series introduced a benzamide that was suggested by docking studies to be oriented toward the entrance of the catalytic site. Probing potential contacts in this area with substituted benzamides eventually revealed **59** as the most potent inhibitor of their study with an IC_50_ = 2.03 µM (Figure 19). Inhibition kinetics suggest **59** to be a non-competitive inhibitor of diphenolase activity and it is assumed the inhibitor may both occupy the active site and hinder the access of the native substrate, but allosteric binding cannot be completely ruled out based solely on these observations. 

The Kolbe group identified a potent hTYR inhibitor, thiamidol (**60**, Figure 19) through a high-throughput screening (HTS) campaign using the Evotec library consisting of 50,000 chemically diverse compounds [79]. Interestingly, thiamidol (**60**) only weakly inhibited mTYR (IC_50_ = 108 µM), but potently inhibited hTYR (IC_50_ = 1.1 µM) despite structural similarity to other known TYR inhibitors. Kinetic studies revealed thiamidol (**60**) to be a competitive inhibitor with a K_i_ of 0.25 µM. With this exciting hTYR selective profile, thiamidol (**60**) was advanced to cell-based studies and, eventually, into a clinical setting. In a melanocyte model, other known TYR inhibitors (e.g., arbutin (**3**), kojic acid (**1**)) showed negligible or weak inhibition of melanin production, whereas thiamidol (**60**) demonstrated potent, reversible inhibition (IC_50_ = 0.9 µM) with activity comparable to what was seen biochemically. The in vivo efficacy of thiamidol (**60**) was evaluated in elderly human subjects by the topical administration of a formula containing 0.2% thiamidol (**60**) twice daily. After four weeks of treatment, age spots were significantly lighter compared to untreated control age spots. By twelve weeks, some age spots were visually indistinguishable from surrounding skin. Since its disclosure, thiamidol (**60**) has been considered the new gold standard for hTYR inhibition.

### 2.17. 2019

**Figure 20:**
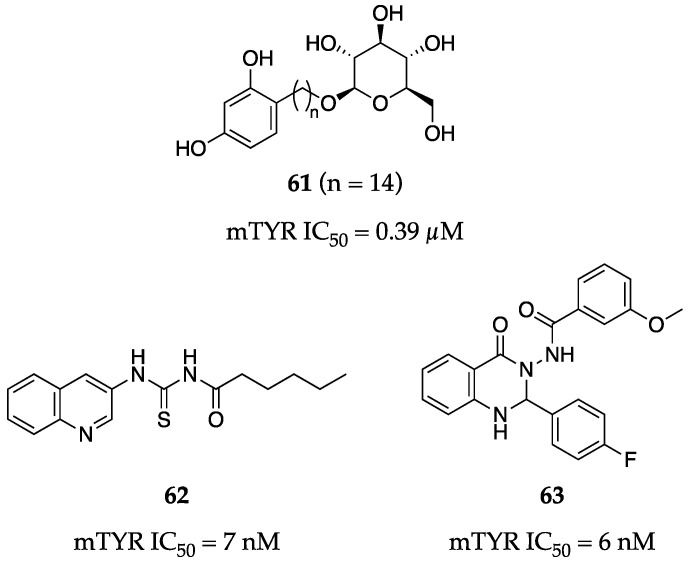
Exemplar inhibitors from 2019 [80,81,82].

Ishioka et al. synthesized a small library of resorcinol alkyl glucosides to investigate a strategy for generating TYR inhibitors with improved water solubility [80]. Resorcinol, a privileged functionality in TYR inhibition, and glucose were tethered together by carbon linkers of various lengths through either Wittig or Horner–Wadsworth–Emmons olefination reactions. The group observed a general trend in which potency and linker length were directly related (i.e., as linker length increased, potency was improved as well), likely due to the glucoside being further away from the catalytic site. Their most potent compound sported a 14-carbon alkyl spacer (**61**, Figure 20) and exhibited a sub-µM IC_50_ value of 0.39 µM.

The Seo group synthesized two series of structurally related quinolines and quinazolinones and found that both series had the potential for potent TYR inhibition [81,82]. Both sets generally demonstrated sub-µM inhibition and both investigations resulted in single-digit nM inhibitors. Exemplars **62** and **63** impressively inhibited mTYR in a non-competitive manner with IC_50_ values of 7 nM and 6 nM, respectively (Figure 20). It is worth noting that the multicomponent reaction used to synthesize the quinazolinone series (e.g., **63**) likely resulted in racemic mixtures, which were not separated prior to biochemical testing. 

### 2.18. 2020

**Figure 21:**
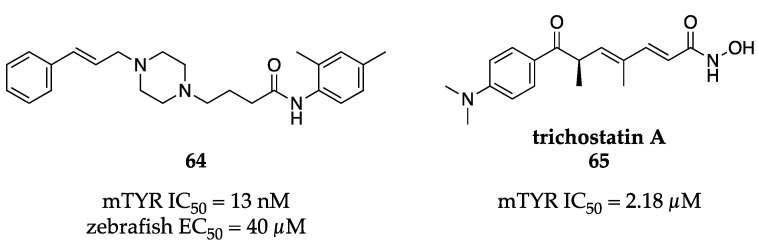
Exemplar inhibitors from 2020 [83,84,85].

Raza et al., also from the Seo group, described a series of non-competitive piperazine butanamides with biochemical anti-tyrosinase activity in the sub-µM to low nM range [83,84]. Their most potent compound (**64**, Figure 21) demonstrated an IC_50_ value of 13 nM against mTYR and was progressed to an in vivo study in zebrafish. Compound **64** caused a significant reduction in pigmentation in the zebrafish model and had an EC_50_ of roughly 40 µM. 

A small set of trichostatins was isolated by Georgousaki et al. from *Streptomyces* sp. CA-129531 and screened for anti-tyrosinase activity [85]. The authors found that trichostatin A (**65**, Figure 21) was 6-fold more potent than kojic acid (**1**) against mTYR with an IC_50_ of 2.18 µM. Based on the activities of close analogs, the hydroxamic acid appeared to be important for inhibition and was potentially the chelating motif. Interestingly, these trichostatins also displayed cytotoxicity in various cancer cells lines with low to sub-µM IC_50_ values.

### 2.19. 2021

**Figure 22:**
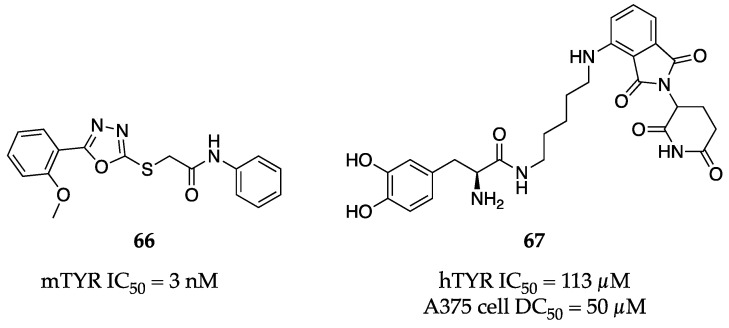
Exemplar inhibitors from 2021 [86,87].

Vanjare et al. synthesized a small library of novel 1,3,4-oxadiazoles, which all demonstrated moderate to low nM inhibitory activity against mTYR [86]. Their most potent compound (**66**, Figure 22) had an IC_50_ = 3 nM in a biochemical assay and significantly diminished melanin synthesis in ⍺-MSH-stimulated cells. Interestingly, **66** also reduced TYR protein levels under the same stimulated conditions. Generally, these oxadiazoles were not cytotoxic up to 50 µg/mL as determined by an MTT assay. 

Proteolysis targeting chimeras (PROTACs) have recently gained significant attention in the medicinal chemistry and pharmaceutical fields. Briefly, a PROTAC is a heterobifunctional molecule capable of binding to a target of interest and recruiting an E3 ligase to mark that target for degradation by the proteosome. Fu et al. developed the first TYR PROTAC using L-dopa as the TYR ligand and thalidomide as the E3 ligase recruiter component [87]. Their initial work utilized kojic acid (**1**) as the TYR ligand, but they found these kojic-acid-based PROTACs to be ineffective in degrading TYR. However, the L-dopa-derived PROTACs were able to induce TYR degradation, albeit at moderate to high concentrations (e.g., 50–100 µM). Their most potent PROTAC (**67**, Figure 22) exhibited a biochemical IC_50_ of 113 µM against hTYR, but a DC_50_ value (i.e., the concentration for 50% protein degradation) of approximately 50 µM when investigated in human A375 melanoma cells. A simple SAR campaign focusing on the L-dopa functionality revealed that both the catechol and the ⍺-amino group are required for activity. Exemplar **67** was progressed to the zebrafish model in which it effectively reduced relative melanin content by roughly 50% at 100 µM, outperforming both L-dopa and kojic acid (**1**) in the same assay. Lastly, selectivity of **67** was assessed against a few receptors of concern—the DOPA receptor and the ⍺1 and β1 adrenergic receptors. Gratifyingly, no degradation of these receptors was observed at 100 µM administration of **67.**

### 2.20. 2022

**Figure 23:**
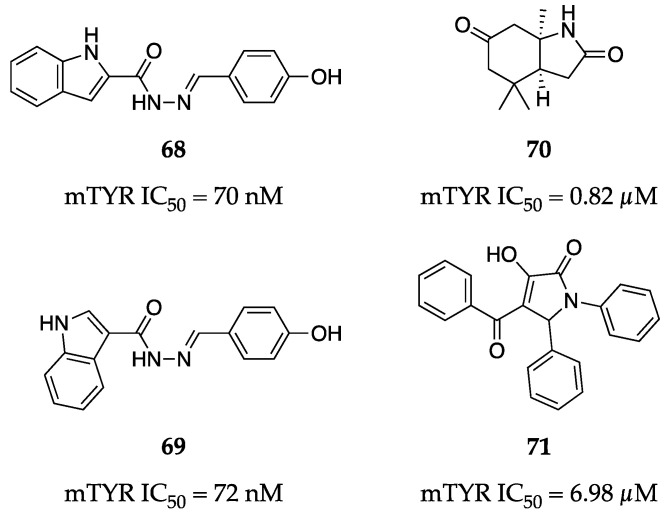
Exemplar inhibitors from 2022 [88,89,90].

A library of twenty-five indole–carbohydrazides was synthesized and evaluated by Iraji et al. for TYR inhibitory activity [88]. The activities of the series ranged from low nM to >100 µM in the enzymatic assay against mTYR. The most potent compounds were a pair of isomers (**68** and **69**, Figure 23) demonstrating equipotent IC_50_ values of 70 nM and 72 nM, respectively. Kinetic studies revealed these to be mixed-type inhibitors. When progressed to evaluation in murine B16F10 melanoma cells, both isomers were able to reduce melanin content in a similar and dose-dependent manner.

Xu et al. isolated four previously undescribed compounds from *Elephantopus scaber* I. and elucidated their structures by means of NMR spectroscopy, electronic circular dichroism (ECD) calculations, and single-crystal X-ray crystallography [89]. Of the four novel compounds, only one exhibited anti-tyrosinase activity (**70**, Figure 23). Alkaloid **70** demonstrated roughly 3.5-fold greater potency against mTYR than arbutin (**3**) with IC_50_ values of 0.82 µM and 2.95 µM, respectively.

Alizadeh et al. designed, synthesized, and evaluated a small library of substituted 3-hydroxy-1H-pyrrol-2(5H)-one analogs against mTYR [90]. Out of the nineteen tested compounds, only four inhibited TYR activity by ≥50% at 50 µM. The most potent compound (**71**, Figure 23) was roughly 2.5-fold more potent than kojic acid (**1**), with an IC_50_ value of 6.98 µM. Kinetic studies revealed **71** to be a mixed-type inhibitor and molecular docking studies suggested binding to be mostly driven by π interactions of the multiple phenyl rings. It is worth noting the chemistry used to synthesize these 3-hydroxy-1H-pyrrol-2(5H)-one analogs likely resulted in a mixture of stereoisomers, which were not separated before testing. Further optimization of the synthetic route or implementation of separation techniques may be warranted to identify the active stereoisomer. 

### 2.21. 2023

**Figure 24:**
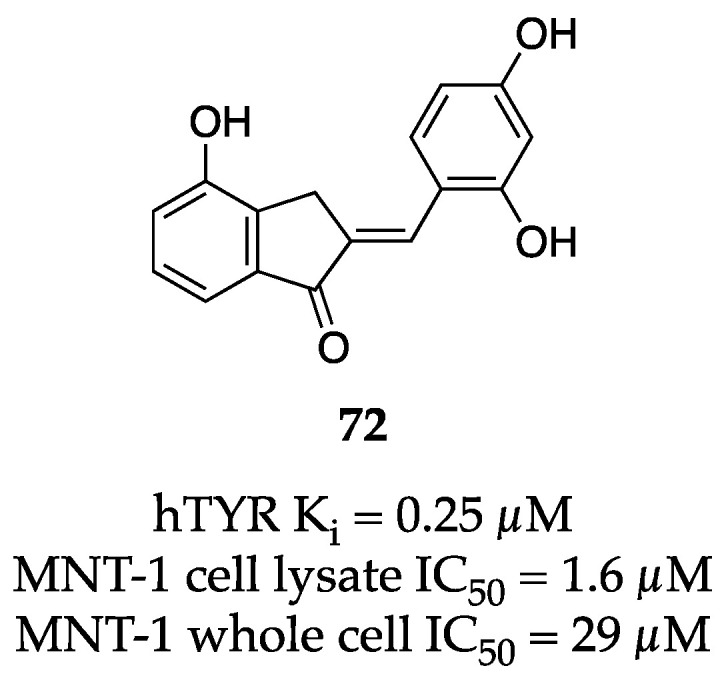
Exemplar inhibitor from 2023 [91].

At the time of this writing, we are halfway through 2023. Despite this, some groups have already reported significant strides in TYR inhibition research. The Haudecoeur group recently published further advances on their aurone lead, which was first disclosed in 2017 as the most potent hTYR inhibitor at that time [91]. In the newest iteration, Haudecoeur et al. have introduced a resorcinol—a privileged motif seen in many other potent TYR inhibitors—and elaborated their SAR campaign on the aurone core. The group prepared a series of thirty-eight resorcinol-based compounds and evaluated their hTYR inhibition activity through both biochemical and cell-based assays. Exemplar **72** demonstrated the best profile out of the set with excellent activity in MNT-1 cell lysates (IC_50_ = 1.6 µM), in MNT-1 whole cells (IC_50_ = 29 µM), and against purified hTYR (K_i_ = 0.25 µM) (Figure 24). Furthermore, **72** demonstrated a partially competitive inhibition mechanism and its activity is comparable to thiamidol (K_i_ = 0.25 µM). Overall, **72** represents a significant contribution to the small portfolio of hTYR inhibitors. 

## 3. Conclusions

The past twenty years of research into the discovery and optimization of inhibitors of TYR, the key enzyme catalyzing the rate-limiting step in melanogenesis, have been a very exciting time rich with novel discoveries. Research groups from across the globe have unearthed chemical matter from varied sources such as plants, fungi, microorganisms, in silico screens, cellular screens, and synthetic libraries. Several groups such as Boissy and Kolbe have been able to take molecules (i.e., **15** and **60**) to the market [41,79]. Based on the diversity and success of research efforts throughout these twenty years, we look forward to the next twenty years of amazing scientific research and discoveries toward effective and safe TYR modulation.

## Data Availability

No new data were created or analyzed in this study. Data sharing is not applicable to this article.
